# Regional clustering and waves patterns due to COVID-19 by the index virus and the lambda/gamma, and delta/omicron SARS-CoV-2 variants in Peru

**DOI:** 10.12688/gatesopenres.13644.2

**Published:** 2023-11-20

**Authors:** Melissa Toyama, Lucía Vargas, Sofía Ticliahuanca, Antonio M Quispe

**Affiliations:** 1 Facultad de Medicina Humana, Universidad Nacional Mayor de San Marcos, Lima, Lima, 15001, Peru; 2 Facultad de Derecho y Ciencia Política, Universidad Nacional Mayor de San Marcos, Lima, Lima, Peru; 3 Facultad de Medicina Humana, Universidad Continental, Huancayo, Junin, 12000, Peru

**Keywords:** COVID-19, SARS-CoV-2, mortality, epidemiology, cluster analysis, Peru

## Abstract

**Background:**

Coronavirus disease 2019 (COVID-19) impact varies substantially due to various factors, so it is critical to characterize its main differences to inform decision-makers about where to focus their interventions and differentiate mitigation strategies. Up to this date, little is known about the patterns and regional clustering of COVID-19 waves worldwide.

**Methods:**

We assessed the patterns and regional clustering of COVID-19 waves in Peru by using the weekly mortality rates for each of the 25 regions as an outcome of interest. We obtained the death counts from the National Informatics System of Deaths and population estimates from the National Registry of Identification and Civil Status. In addition, we characterized each wave according to its duration, peak, and mortality rates by age group and gender. Additionally, we used polynomial regression models to compare them graphically and performed a cluster analysis to identify regional patterns.

**Results:**

We estimated the average mortality rate at the first, second, and third waves at 13.01, 14.12, and 9.82 per 100,000 inhabitants, respectively, with higher mortality rates among elders and men. The patterns of each wave varied substantially in terms of duration, peak, impact, and wave shapes. Based on our clustering analysis, during the first wave caused by the index virus, the 25 regions of Peru presented six different wave patterns. However, the regions were clustered in two different wave patterns during the second and third, caused by alpha/lambda/delta and omicron.

**Conclusions:**

The propagation of severe acute respiratory syndrome coronavirus 2 (SARS-COV-2) variants behaved in Peru with varying wave patterns and regional clustering. During the COVID-19 pandemic, the weekly mortality rates followed different spatiotemporal patterns with solid clustering, which might help project the impact of future waves of COVID-19.

## Introduction

Coronavirus disease 2019 (COVID-19) was first reported in Wuhan, China, in December 2019 ^1^ and spread rapidly throughout the world. Its arrival in Latin America was registered on February 26, 2020, in Brazil ^2^, and the first case in Peru was confirmed just nine days later, on March 6 ^3^. Peru became one of the first Latin American countries to implement quarantine and other restrictive measures; however, the spread of the index virus did not stop across Peru ^4^. One year later, Peru accumulated over 210,000 deaths due to COVID-19, leading the list of countries with the highest mortality rates worldwide in the first and second waves ^5^.

Peru has registered three COVID-19 waves, dominated primarily by the index virus and the lambda/gamma, and delta/omicron SARS-CoV-2 variants, respectively ^6^. During these waves, some regions suffered waves of catastrophic magnitudes, such as the first wave of the region of Loreto, with the capital city of Iquitos ^7^ reporting the highest seroprevalence worldwide in July 2020 ^8^. Peru reported the beginning of the second wave in November 2020, which soon became the deadliest wave in Latin America again, mainly because of the rapid spread of the gamma and lambda variants ^9^, and the many lessons not learned during its first wave ^10^. The third wave in Peru started slowly, with record case numbers increasing explosively in January 2022, reaching a peak over five times higher than the peak observed at the second wave. The third wave was characterized by the initial predominance of delta and the explosive increase of cases due to the introduction of omicron BA.1, but had significantly lower mortality than previous waves.

Now that the third wave is currently ending in Latin America, it is crucial to collect the many lessons learned from this experience to inform the decision-making in future waves. Like many low-middle-income countries, Peru has health systems with inequalities that disproportionately impact its different regions and provinces ^11^. Certainly, we can take many lessons from previous waves and further understand how COVID-19 spread across countries’ regions. This information may help adapt public health interventions, maximize their impact, and adjust to their common regional characteristics ^12^ and different demographics ^13^. Unfortunately, very little has been described in the literature on this critical subject. This study seeks to fill this scientific knowledge gap by assessing the all-cause mortality per epidemiological week as the outcome of interest, since it has been reported consistently as the most reliable tracer of the impact of the COVID-19 pandemic in low-middle-income countries ^14^. Thus, we analyzed the weekly mortality rate to characterize each of the three COVID-19 waves that affected Peru at the regional level and assessed their patterns and regional clustering.

## Methods

### Study design and population

We conducted a cross-sectional study to characterize and compare the first, second, and third waves of COVID-19 in each region of Peru. Peru’s territory encompassed 25 regions (“departamentos”), which are subdivided into 196 provinces (“provincias”) and 1,869 districts (“distritos”). For this study, we grouped all the provinces of Lima as the Lima region and included the constitutional province of Callao as an independent region. Consequently, we used the same classification of regions used by the study data sources in our analysis.

### Study outcome and data sources

We used the weekly mortality rate as the study outcome. At the national and regional levels, we calculated the weekly mortality rate by multiplying the accumulated death counts per epidemiological week by 100,000 and dividing the product by the estimated annual population. We obtained the death counts, population estimates, variants distributions, and region's geographical boundaries using open data curated from the government of Peru. We described the metadata and links from each of these sources in Table 1. We obtained the death counts from all causes from the National System of Deaths (SINADEF), which is updated daily at the Unique National Health Information Repository (REUNIS) ^15^. We obtained the annual estimated population at the regional and national level from the National Institute of Statistics and Informatics (INEI) ^16^, registering Peruvian citizens and accurately allocating their residence region. We obtained the weekly distribution of SARS-CoV-2 variants circulating in Peru from the Peruvian National Institute of Health (INS) ^17^. Finally, we got the map shapefiles from the Peruvian Ministry of the Environment (MINAM) ^18^.

**Table 1.** Metadata for the datasets used in the research article.

Name	Provider	Year	Format	Variable	Source
Peruvian Population 2021	National Institute of Statistics and Informatics (INEI)	2021	Comma- separated values (CSV)	Continuous	https://www.datosabiertos.gob.pe/dataset/ poblaci%C3%B3n-peru
National Deaths Informatics System (SINADEF)	Ministry of Health (MINSA)	2022	Comma- separated values (CSV)	Continuous	https://www.datosabiertos.gob.pe/dataset/ informaci%C3%B3n-de-fallecidos-del-sistema- inform%C3%A1tico-nacional-de-defunciones-sinadef- ministerio
Genomic sequencing of the SARS-CoV-2 virus in Peru	National Institute of Health (INS)	2022	Power Bi Dashboard	Continuous	https://web.ins.gob.pe/es/covid19/secuenciamiento- sars-cov2
Peru regional boundaries	Ministry of the Environment (MINAM)	2007	Shapefile	Continuous	https://geoservidorperu.minam.gob.pe/geoservidor/ archivos/download/Limite_departamental.rar

**Legend:** The "Format" column indicates the extension of the data. The "Variable" column reports the variable type of the data. Finally, the "Source" column presents the links to each of the datasets used in our study.

### Statistical analysis

We performed a descriptive analysis to characterize the COVID-19 waves by calculating the weekly mortality rates at the national and regional levels. We used the epidemiological week in which the Peruvian Ministry of Health confirmed the first COVID-19 death to set the beginning of each first wave, and the end of the third wave as the point at which the weekly mortality rate returned to pre-pandemic levels. We performed a graphical analysis of the weekly mortality rates at the national and regional levels by using the “ggplot2” package ^19^. We fit a segmented regression model with unknown break-points to assess the end of the first and second waves, which we defined as the point where the trend in the weekly mortality rates changed from negative to positive. For this purpose, we used the methodology developed by Muggeo VM ^20^ and the package "segmented" designed by the same author. Next, we calculated and described the mortality variability between women and men and among the age groups of 0 to 19, 20 to 59, and 60 years old and older to assess whether the demographics affected the comparability of the mortality among regions. After this verification, we compared the unstandardized mortalities instead of the standardized mortalities adjusted by age and gender. To compare the duration of each wave, we quantified the "time from the beginning to the peak" and the "total duration" of each wave, both in epidemiological weeks. Also, we compared the peak of each wave (measured as the higher weekly mortality rate), the wave onset week, and the wave last week, which we tabulated as a heat map. Finally, we characterized the pattern of each regional wave and performed a K-means clustering analysis to assess typical patterns during Peru's first, second, and third waves separately using the regional weekly mortality as the study outcome. During the K-means clustering analysis, we used the elbow method to determine how many clusters minimized the intra-cluster variance and maximized the inter-cluster variance. And, to confirm the findings of this analysis, we used the silhouette method, which measures the quality of clustering by calculating the average silhouette, which was used to graphically identify the optimal number of clusters by plotting the highest value of the average silhouettes. We used R 3.6.1 (R Foundation for Statistical Computing, Vienna, Austria) and R Studio 1.2.5001 (Free Software Foundation, Inc., Boston, MA) for the statistical analysis and the QGIS program 3.22 to elaborate the maps with the clustering analysis results.

## Results

### Duration of the first, second, and third COVID-19 waves in Peru

**Figure 1.**
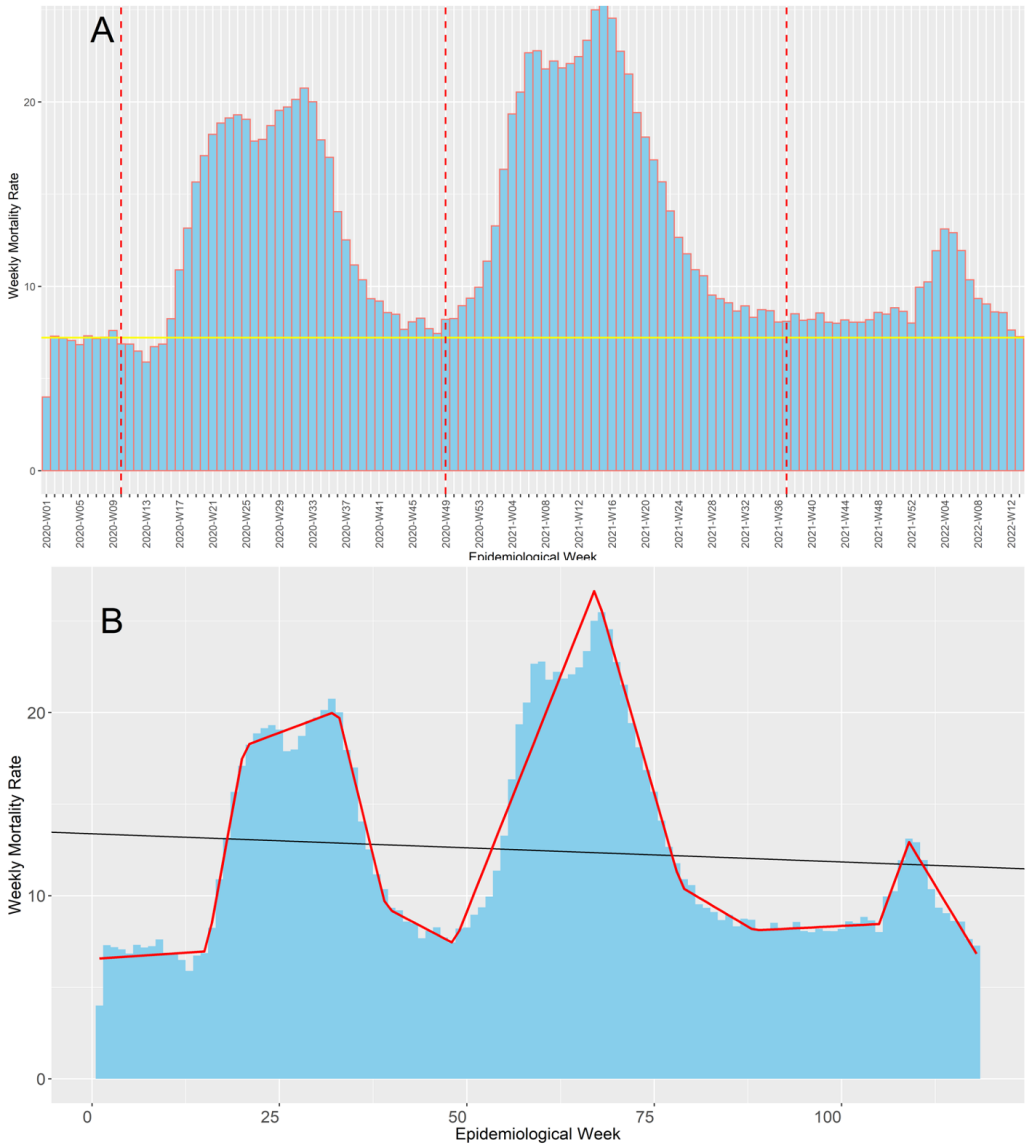
Evolution of the COVID-19 weekly mortality rates* during Peru's first, second, and third COVID-19 waves. The figure shows the evolution of the weekly mortality deaths (death counts per week /100,000 inhabitants of Peru) along the pandemic in Peru ( **Figure 1A**) contrasted with the baseline mortality pre-pandemic (continuous yellow line) and the beginning epidemiological week of the first, second, and third COVID-19 waves in Peru (red dot lines). Also, it shows the trends estimated in our segmented regression analysis (continuous red line), which we used to determine when each wave started ( **Figure 1B**).

**Figure 2.**
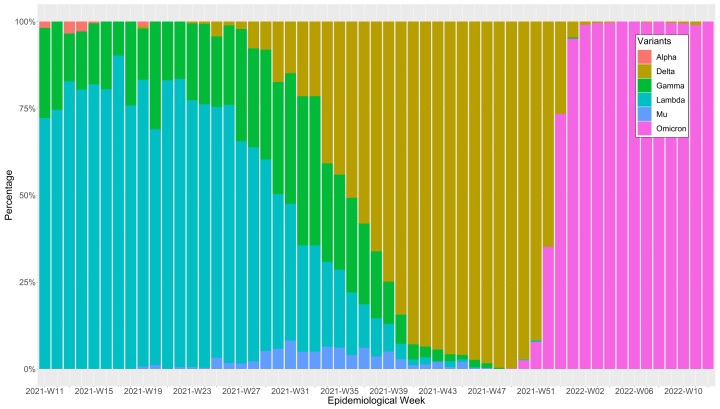
SARS-CoV-2 variants percentages distribution by epidemiological week during Peru's first, second, and third waves of COVID-19. Weekly mortality rates are expressed in death counts per week /100,000 inhabitants of Peru.

The Ministry of Health confirmed the first death by COVID-19 at the epidemiological week 10 of 2020, and up to the epidemiological 13-2022, Peru suffered three COVID-19 waves ( Figure 1A). Based on our segmented regression analysis ( Figure 1B), this first wave most likely lasted 39 weeks ending at the epidemiological week 48 of 2020, which consequently was the epidemiological week with the lowest weekly mortality rate before the second wave started. On the other hand, we found that the second wave was the deathlier and the longest among the three waves, with 41 weeks from the epidemiological week 49-2020 until the epidemiological 36-2021. The end of the second wave coincides with the epidemiological week in which delta became the predominant SARS-CoV-2 variant in Peru, representing over 50% of them ( Figure 2). However, the weekly mortality rate increment was minimal until it became explosive when omicron displaced delta and became the predominant variant in the epidemiological week 52-2022. Regardless, the third wave was the less deathly and the shortest one, with an overall duration of 29 weeks from the epidemiological week 37-2021 to the 13-2022. Consequently, the epidemiological week 13-2022 was when the weekly mortality rates returned to pre-pandemic levels.

### Mortality during the first, second, and third COVID-19 waves in Peru

In the first three waves of the COVID-19 pandemic and for 109 weeks, Peru accumulated 514,006 deaths from all causes. Given that the pandemic started in the 10th epidemiological week and the first epidemiological week included the last three days of 2019, we calculated the weekly baseline mortality before the first wave as the mean mortality per week during epidemiological weeks two to nine, which was 7.21 ± 0.22 deaths per 100,000 people. Consequently, we calculated the excess deaths during the first three waves as 258,106 deaths, including 88,273 in the first wave, 145,401 in the second wave, and 24,432 in the third one. Based on these counts, we calculated that the excess deaths represented 49%, 60%, and 26% of all the deaths in the first, second, and third waves, respectively. On the contrary, we observed that the mortality ratio between the adults 60 years old and older and the adults 20 to 59 years-old progressed from 13.1 to 13.4 and 16.2 in the first, second, and third waves. Overall, the second wave was deathlier than the first one, and the first wave was deathlier than the third one ( Table 2).

**Table 2.** National mortality variability during the COVID-19 pandemic in Peru.

	Deaths counts			Mortality		
	1st Wave	2nd Wave	3rd Wave	1st Wave	2nd Wave	3rd Wave
**Total deaths**	180 014	241 551	92 441	553.45	742.64	284.21
**Excess deaths**	88 273	145 401	24 432			
**By gender**						
** Women**	70 337	103 703	43 207	427.97	647.59	269.81
** Men**	109 677	137 848	49 234	677.40	834.81	298.16
**By age groups**						
** 0 to 19 y. o.**	5658	8553	5263	53.43	74.65	45.94
** 20 to 59 y. o.**	43 249	64 079	20 808	241.68	364.07	118.22
** 60 or over y. o.**	131 022	168 864	66 370	3164.45	4869.22	1913.36

**Legend:** Mortality is expressed in death counts per wave/100,000 inhabitants of Peru.

### Variability of the mortality rate across regions by wave, age, and gender

**Figure 3.**
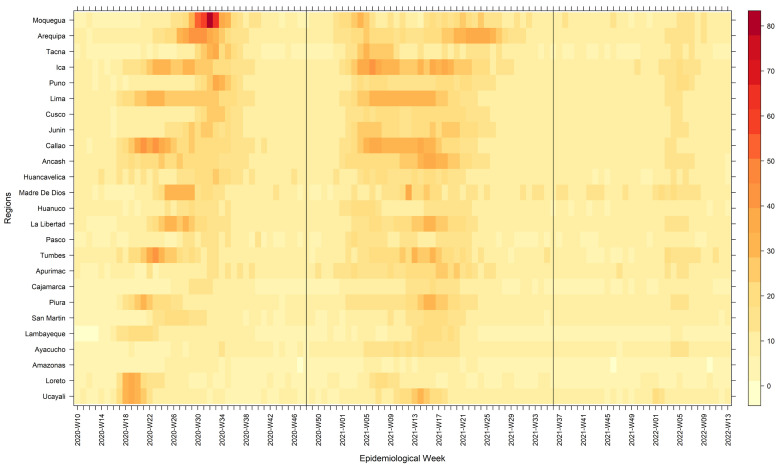
Heat map of the weekly mortality rates by region during Peru's first, second, and third waves of COVID-19. Weekly mortality rates are expressed in death counts per week /100,000 inhabitants of Peru.

Overall, at the national level, higher mortality was recorded during the second wave (743 deaths per 100,000 inhabitants), followed by the first wave (553 deaths per 100,000 inhabitants) and the third wave (284 deaths/per 100,000 inhabitants). At the regional level, we observed that during the first waves, the three regions with the highest mortality overall were Callao (750 deaths per 100,000 inhabitants), Lima (750 deaths per 100,000 inhabitants), and Moquegua (681 deaths per 100,000 inhabitants). During the second wave, the regions with the highest mortality overall were Ica (1035 deaths per 100,000 inhabitants), Arequipa (927 deaths per 100,000 inhabitants), and Lima (894 deaths per 100,000 inhabitants). And during the third wave, the regions with the highest mortality overall were Madre de Dios (395 deaths per 100,000 inhabitants), Ica (349 deaths per 100,000 inhabitants), and Arequipa (341 deaths per 100,000 inhabitants). Furthermore, we observed high variability of the weekly mortality weeks among waves and, within each wave, across regions ( Figure 3). Nevertheless, it was consistently higher among men than women and adults 60 years old and older compared to other age groups ( Table 3). At the regional level, we observed that during the first, second, and third waves, the mortality rate among men was higher than among women in all regions except for Arequipa during the third wave. Likewise, the mortality among the people 60 years old and older was higher than those aged 20 to 59 years old and 0 to 19 years old in all three waves, with the elders' age group consistently having the highest mortality rates. During the first wave, the region of Huancavelica had the most increased mortality among people 60 years old and older, with 4,413 deaths per 100,000 inhabitants. During the second wave, Ica was the region with a higher mortality rate, with 6,131 deaths per 100,000 inhabitants. And during the third wave, Huancavelica was again the region with a higher mortality rate, with 2,673 deaths per 100,000 inhabitants. Madre de Dios had a higher mortality rate among the people 20 to 59 years old age group, with 361 deaths per 100,000 inhabitants during the first wave. During the second wave, Ica registered the higher mortality, with 544 deaths per 100,000 inhabitants, and during the third wave, Madre de Dios reported the higher mortality, with 313 deaths per 100,000 inhabitants. Among people in the 0 to 19 years old age group, Madre de Dios had the higher mortality rate during the first, second, and third wave, with 161,226, and 161 deaths per 100,000 inhabitants, respectively.

**Table 3.** Regional mortality variability by age and gender during COVID-19 pandemic in Peru.

Region	Total mortality			Female mortality			Male mortality			Female/male mortality ratio			0 to 19 years old mortality			20 to 59 years old mortality			60 years old and older mortality		
	W1	W2	W3	W1	W2	W3	W1	W2	W3	W1	W2	W3	W1	W2	W3	W1	W2	W3	W1	W2	W3
Callao	750	852	311	551	772	290	960	1013	331	0.57	0.76	0.88	47	60	34	324	444	128	3505	5056	1840
Lima	701	894	305	518	785	298	898	1003	311	0.58	0.78	0.96	50	56	32	281	424	112	3373	5327	1926
Moquegua	681	720	305	510	582	267	830	870	344	0.61	0.67	0.78	30	86	40	256	315	101	3467	4122	1873
Ica	676	1035	349	532	904	332	819	1168	365	0.65	0.77	0.91	43	80	48	319	544	146	4083	6131	2173
Arequipa	651	927	341	498	823	337	809	1035	344	0.62	0.80	0.98	48	82	48	242	445	134	3533	5325	2033
Ancash	605	852	328	489	743	303	719	960	352	0.68	0.77	0.86	66	89	52	233	371	128	3588	5682	2197
Tumbes	586	730	300	497	568	248	663	897	352	0.75	0.63	0.70	65	113	59	285	357	132	3624	5584	2336
Huancavelica	535	601	265	503	576	264	566	632	265	0.89	0.91	1.00	99	90	51	253	309	116	4413	5987	2673
La Libertad	523	734	306	411	658	291	636	807	319	0.65	0.82	0.91	54	79	52	220	344	127	3014	4859	2044
Madre de Dios	513	695	395	373	476	250	621	945	556	0.60	0.50	0.45	161	226	161	361	534	313	2784	4725	2393
Junín	512	789	299	422	682	279	602	897	318	0.70	0.76	0.88	75	84	51	239	406	127	3268	5696	2212
Piura	472	679	290	363	594	260	580	762	319	0.63	0.78	0.82	39	77	53	212	337	125	3007	4728	2045
Puno	461	670	310	381	579	289	543	757	330	0.70	0.76	0.88	71	85	44	219	330	131	2753	4826	2327
Cusco	447	718	298	391	639	288	502	796	308	0.78	0.80	0.94	78	113	61	204	337	133	3033	4701	1932
Ucayali	431	588	283	346	483	263	509	686	304	0.68	0.70	0.87	61	110	81	253	340	164	3111	4022	1857
Huánuco	423	568	254	378	491	242	466	644	267	0.81	0.76	0.91	61	85	57	201	270	119	3544	4345	1871
Apurímac	399	736	314	385	669	297	412	809	331	0.93	0.83	0.90	59	86	52	160	318	136	3064	5827	2401
Tacna	397	622	240	291	485	215	501	753	265	0.58	0.64	0.81	39	60	29	180	335	100	2096	4078	1729
San Martín	385	543	256	303	452	227	459	644	286	0.66	0.70	0.79	49	95	57	216	287	132	2850	4317	1980
Loreto	372	397	185	282	340	166	455	457	204	0.62	0.74	0.81	56	101	64	240	261	113	2835	2903	1311
Lambayeque	357	412	137	236	335	122	485	466	152	0.49	0.72	0.8	16	33	26	176	222	63	1884	2281	793
Pasco	349	522	229	312	446	221	384	605	236	0.81	0.74	0.94	85	67	55	185	309	112	2255	4274	1893
Ayacucho	292	565	264	260	533	262	322	599	266	0.81	0.89	0.98	47	87	54	131	264	112	2220	4853	2267
Cajamarca	284	460	203	232	418	193	336	498	213	0.69	0.84	0.91	36	68	44	128	209	83	2229	3510	1551
Amazonas	210	279	110	163	242	109	254	311	111	0.64	0.78	0.98	33	58	35	113	152	50	1700	1972	799

**Legend:** Mortality is expressed as the total death counts from all causes per 100,000 inhabitants; W1, wave 1; W2, wave 2; W3, wave 3.

### Regional cluster analysis

**Figure 4.**
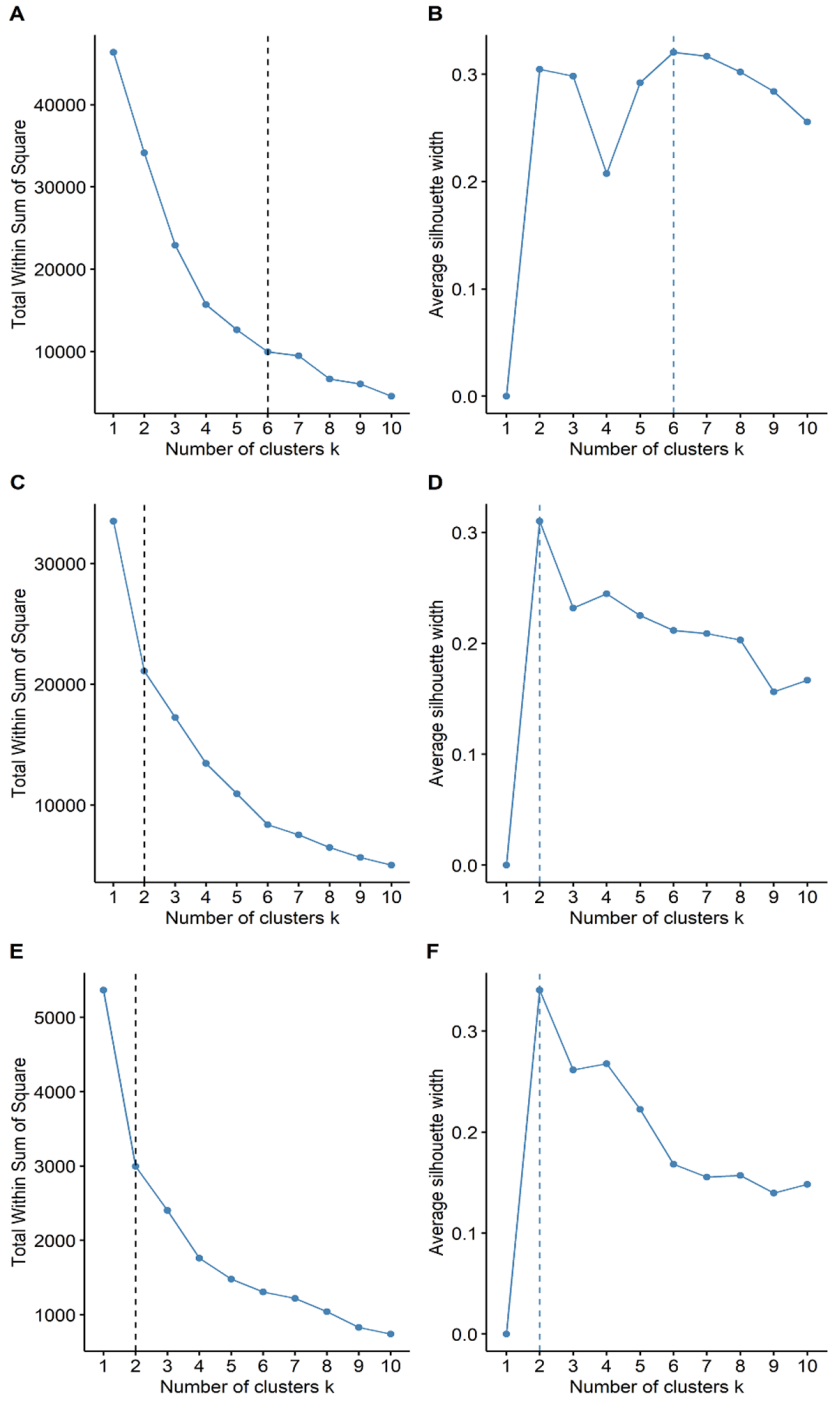
Elbow ( **A**) and Silhouette ( **B**) analysis according to the weekly mortality rate during the first, second, and third waves of COVID-19 in Peru. Elbow ( **A**) and Silhouette ( **B**) clustering analysis during the first wave; Elbow ( **C**) and Silhouette ( **D**) clustering analysis during the second wave; and Elbow ( **E**) and Silhouette ( **F**) clustering analysis during the first wave.

In our cluster analysis, we observed that the spread of COVID-19 across the regions of Peru followed different wave patterns ( Figure 4) in both mortality ( Table 4) and duration ( Table 5). We found six clusters of regions with varying wave patterns in the first wave, either by using the Elbow method ( Figure 4A) or the Silhouette method ( Figure 4B). During the first wave, the regions of Lima and Callao had two of the deathlier and early waves, both beginning in epidemiological week 10. In contrast, Moquegua was the region with the latest onset with a first wave that started at the epidemiological week 16. During the second wave, we characterized two clusters with different region wave patterns ( Figure 4C and Figure 4D). As for the third wave, we also found that the regions of Peru could be classified into two different clusters ( Figure 4E and Figure 4F). 

**Table 4.** Mortality during the first, second, and third wave in Peru.

Region	Basal weekly mortality	Cumulative mortality			Wave excess deaths			Peak´s weekly mortality			Clusters		
		Wave 1	Wave 2	Wave 3	Wave 1	Wave 2	Wave 3	Wave 1	Wave 2	Wave 3	Wave 1	Wave 2	Wave 3
Callao	7.50	697	867	238	427	492	66	40	37	15	1	1	1
Lima	7.25	692	919	141	395	520	47	29	34	15	1	1	1
Ica	8.75	657	1005	229	307	568	63	31	40	19	1	1	1
Ancash	8.88	545	712	361	226	375	59	24	38	16	1	1	1
Tumbes	8.88	518	741	213	216	262	44	40	33	19	1	1	1
Arequipa	8.75	630	902	211	280	465	54	43	39	17	3	1	1
La Libertad	7.38	470	655	282	205	331	76	29	31	14	3	1	1
Junín	8.38	473	784	197	155	349	38	26	27	13	5	1	1
Piura	6.38	409	655	236	186	336	83	30	31	15	2	2	1
Ucayali	7.50	402	558	194	110	183	44	39	29	23	2	2	1
Madre de Dios	9.25	484	728	185	123	210	65	32	35	23	3	2	1
Huánuco	7.88	402	503	195	87	157	14	19	22	12	4	2	1
Apurímac	7.00	341	569	363	96	324	111	15	28	14	4	2	1
San Martín	6.38	329	470	249	106	190	58	23	21	11	4	2	1
Ayacucho	5.75	229	629	124	34	273	49	13	20	13	4	2	1
Cusco	9.00	441	739	136	72	244	19	26	23	13	5	2	1
Huancavelica	9.63	427	657	171	129	118	-12	25	23	13	5	2	1
Puno	8.38	390	639	271	105	237	53	35	21	21	5	2	1
Tacna	6.63	341	629	136	122	251	37	37	31	16	5	2	1
Moquegua	7.50	589	650	281	372	305	71	78	32	17	6	2	1
Loreto	4.75	359	262	226	164	120	46	36	19	12	2	2	2
Lambayeque	6.75	312	407	82	89	9	-26	23	23	8	2	2	2
Pasco	7.25	283	437	241	44	147	2	16	19	11	4	2	2
Cajamarca	5.38	245	434	150	57	165	32	16	19	10	4	2	2
Amazonas	4.25	178	240	74	25	45	-20	12	10	5	4	2	2

**Legend:** Mortality is expressed as the total death counts from all causes per 100,000 inhabitants.

**Table 5.** Duration of the first, second, and third wave in Peru.

Region	Start (epidemiological week)			End (epidemiological week)			Time to peak (weeks)			Duration (weeks)			Clusters		
	Wave 1	Wave 2	Wave 3	Wave 1	Wave 2	Wave 3	Wave 1	Wave 2	Wave 3	Wave 1	Wave 2	Wave 3	Wave 1	Wave 2	Wave 3
Callao	10	46	43	45	42	12	11	14	13	36	50	23	1	1	1
Lima	10	51	53	50	52	12	12	9	2	41	55	13	1	1	1
Ica	10	50	47	49	46	11	14	9	10	40	50	19	1	1	1
Ancash	11	47	32	46	31	13	13	21	25	36	38	34	1	1	1
Tumbes	12	46	47	45	46	13	11	20	8	34	54	19	1	1	1
Arequipa	11	51	48	50	47	12	20	25	9	40	50	18	3	1	1
La Libertad	11	47	38	46	37	13	14	21	18	36	44	28	3	1	1
Junín	10	48	47	47	46	13	21	10	9	38	52	19	5	1	1
Piura	10	45	42	44	41	12	11	23	14	35	50	24	2	2	1
Ucayali	10	49	46	48	45	11	9	18	7	39	50	20	2	2	1
Madre de Dios	11	50	53	49	52	13	17	15	2	39	56	13	3	2	1
Huánuco	12	52	43	51	42	13	17	5	13	40	44	23	4	2	1
Apurímac	13	48	30	47	29	13	26	23	28	35	35	36	4	2	1
San Martín	10	45	36	44	35	12	15	24	17	35	44	30	4	2	1
Ayacucho	10	44	53	43	52	12	24	21	3	34	62	13	4	2	1
Cusco	10	51	53	50	52	13	24	16	3	41	55	13	5	2	1
Huancavelica	13	44	47	43	46	13	20	24	9	31	56	19	5	2	1
Puno	11	45	40	44	39	13	22	14	17	34	48	26	5	2	1
Tacna	12	45	49	44	48	11	21	13	8	33	57	15	5	2	1
Moquegua	16	45	38	44	37	12	16	12	19	29	46	28	6	2	1
Loreto	10	51	28	50	27	12	12	9	24	41	30	38	2	2	2
Lambayeque	10	43	49	42	48	12	10	25	8	33	59	16	2	2	2
Pasco	13	46	33	45	32	11	16	10	20	33	40	33	4	2	2
Cajamarca	12	47	44	46	43	13	18	22	13	35	50	22	4	2	2
Amazonas	12	48	41	47	40	10	17	22	7	36	46	22	4	2	2

**Legend:** Mortality is expressed as the total death counts from all causes per 100,000 inhabitants.

### Regional patterns during the first wave caused by the index virus

**Figure 5.**
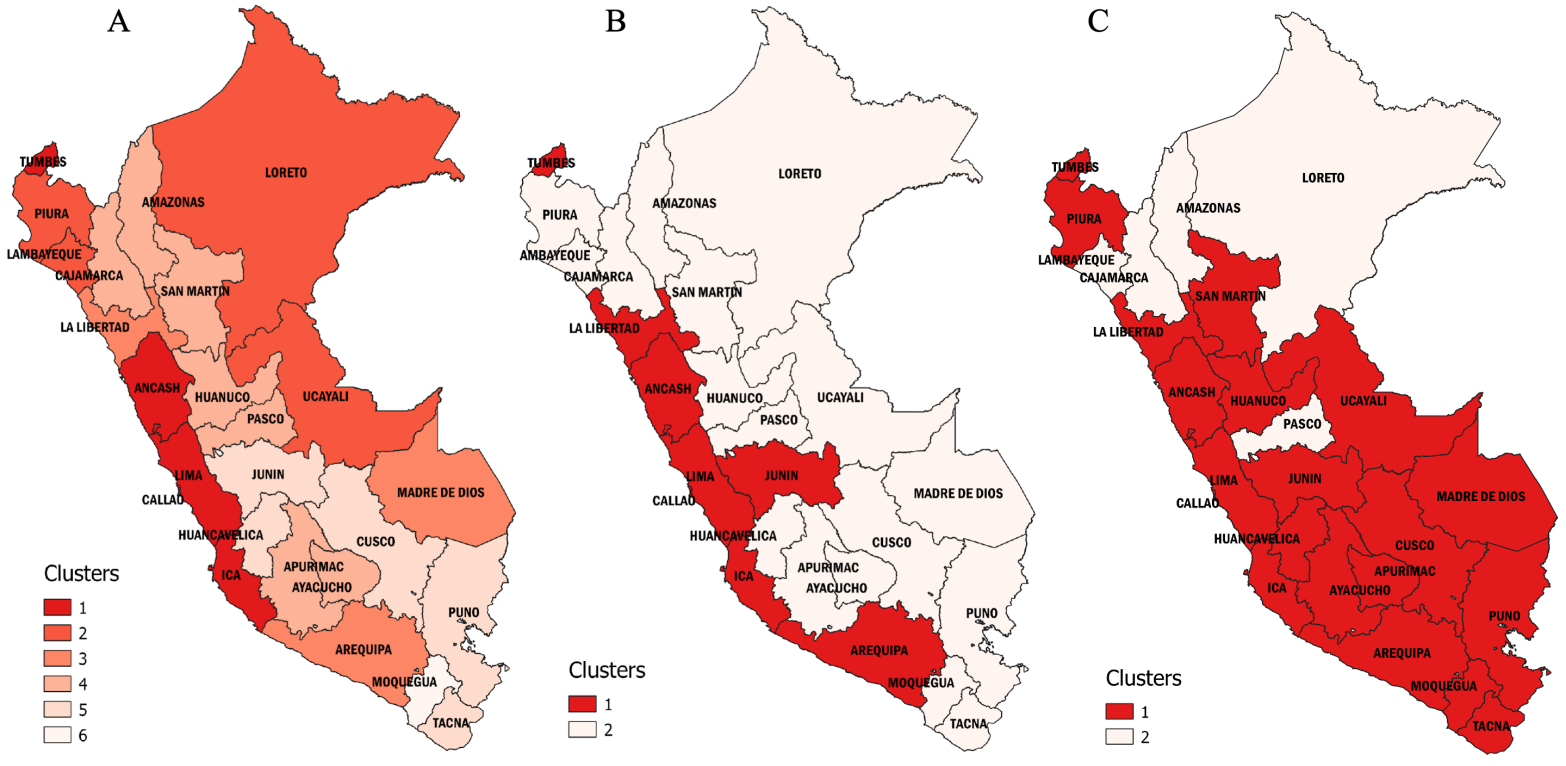
Regional clustering according to the weekly mortality rate during Peru's first, second, and third waves of COVID-19. Clusters of regions based on the weekly mortality rates (death counts per week /100,000 inhabitants) during the first ( **A**), second ( **B**), and third ( **C**) waves of COVID-19 in Peru.

**Figure 6.**
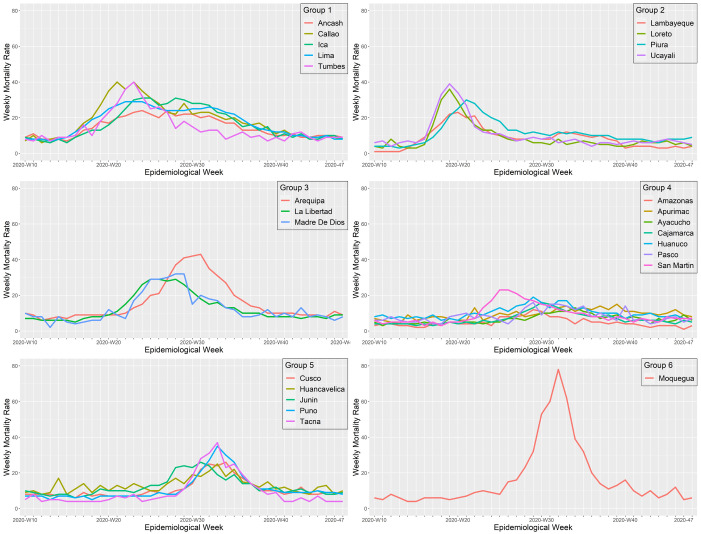
Regional wave patterns of the weekly mortality rate during the first wave of COVID-19 in Peru. Mortality is expressed as the total death counts from all causes per 100,000 inhabitants.

The first wave of Peru started in Lima, the region that reported the first COVID-19 cases and the first COVID-19 death in the country. Lima showed a similar wave pattern to its surrounding regions, including Ancash in the North, Callao in the East, and Ica in the South. Another region that showed a similar wave pattern to Lima was Tumbes on the northern border of Peru, frontier with Ecuador, which implies that COVID-19 cases may also have entered Peru from Ecuador at the beginning of its first wave ( Figure 5A). During the first wave, the regions of Peru exhibited six different wave patterns ( Figure 6). Overall, all the regions from "cluster one" exhibited a first wave characterized as the earliest and longer lasting, with a mortality rate that did not exceed 40 deaths per 100,000 inhabitants. The first wave spread from Tumbes to Piura, Piura to Lambayeque by the Panamericana highway, and from Lima to Loreto and Ucayali by air traffic. All from "cluster two," these regions exhibited an early and short duration first wave, with a peak weekly mortality rate of 39 deaths per 100,000 inhabitants. Then, the first wave seems to have spread to the nearby regions, including Arequipa from Ica, La Libertad from Ancash, and Madre de Dios from Ucayali. All the regions from "cluster three" exhibited a short duration and intermediate first wave, with a maximum mortality rate of 43 deaths per 100,000 inhabitants. Then, the first wave moved to the highlands, impacting the regions of Amazonas, Apurímac, Ayacucho, Cajamarca, Huánuco, Pasco, and San Martín. The "cluster four" showed a moderate first wave with peaks that did not exceed a mortality rate of 23 deaths per 100,000 inhabitants. Finally, the first wave spread to the southern regions of Peru, impacting the regions of Cusco, Huancavelica, Junín, Puno, and Tacna; all these regions from cluster three had the latest and shorter first waves, with a maximum mortality rate of 37 deaths per 100,000 inhabitants. Among the southern regions of Peru, Moquegua behaves as a stand-alone cluster (cluster six). Despite having one of the latest first waves, Moquegua had the higher peak among all the regions of Peru, with a weekly mortality rate of 78 deaths per 100,000 inhabitants. Moquegua's peak during the first wave was also the highest across all regions and the three waves.

### Regional patterns during the second wave, a wave that shifted from alpha to lambda and gamma predominance

**Figure 7.**
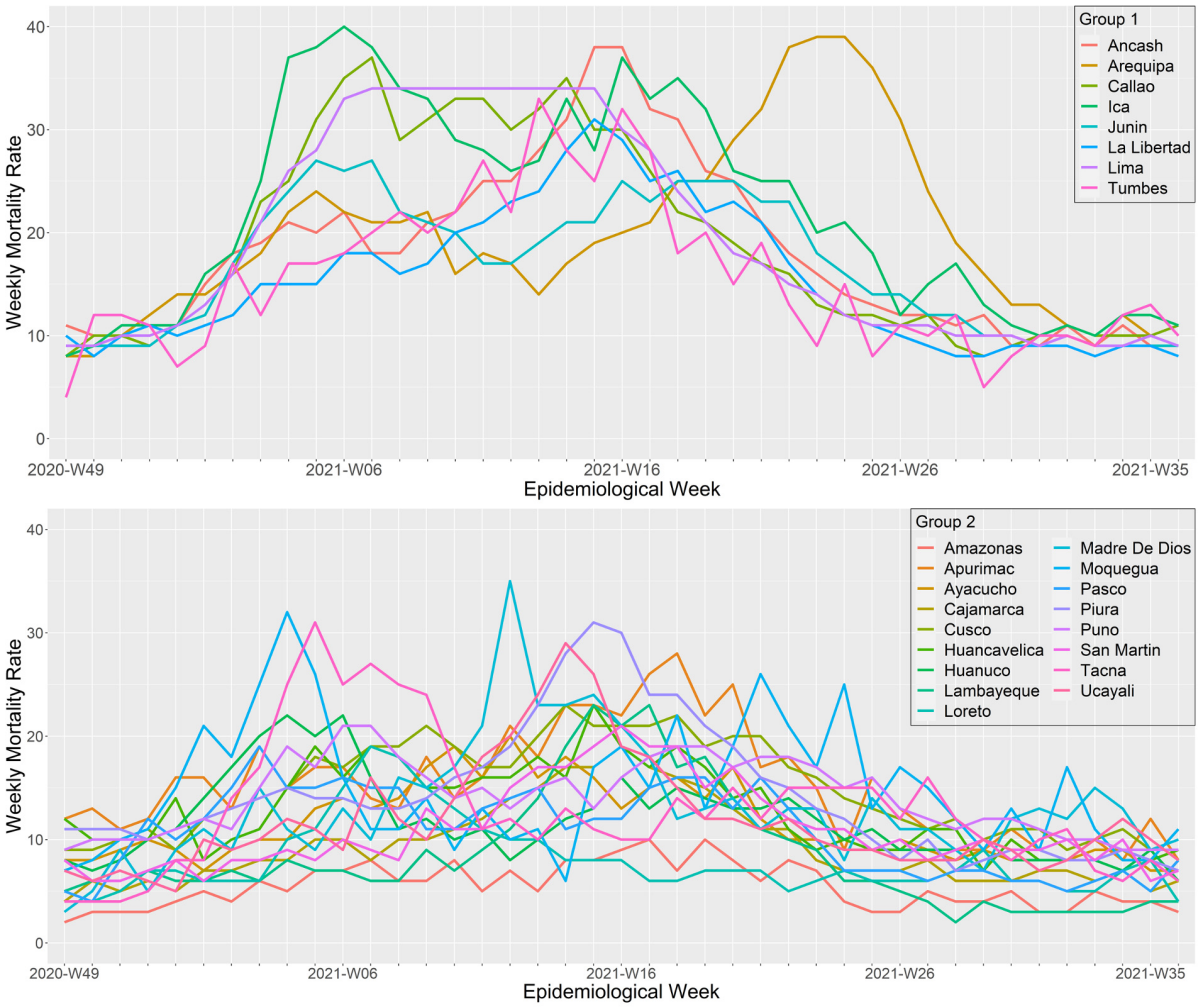
Regional wave patterns of the weekly mortality rate during the second wave of COVID-19 in Peru. Mortality is expressed as the total death counts from all causes per 100,000 inhabitants.

During the second wave, the regions of Peru exhibited two different wave patterns ( Figure 5B and Figure 7). The first cluster was composed by the regions of Ancash, Arequipa, Callao, Ica, Junín, La Libertad, Lima, and Tumbes. During the second wave, these regions had deathlier second waves with higher weekly mortality rates (peak mortality range: 27 to 40 deaths per 100,000 inhabitants). Callao and Ancash had the earliest onset (epidemiological weeks 46 and 47 of 2020, respectively) and Ica the deathlier second wave, with a peak of 40 deaths per 100,000 inhabitants. The second cluster included the regions of Amazonas, Apurimac, Ayacucho, Cajamarca, Cusco, Huancavelica, Huánuco, Lambayeque, Loreto, Madre de Dios, Moquegua, Pasco, Piura, Puno, San Martin, Tacna, and Ucayali. Overall, this cluster had some regions with the faster peak second waves (Pasco, Tacna, Loreto Huánuco, and Moquegua, all with a time to peak below 10 weeks) and some regions with the lower mortality (Amazonas, Pasco, Loreto, and Cajamarca, all with mortalities at the wave peak bellow 20 deaths per 100,000 inhabitants).

### Regional patterns during the third wave, a wave that shifted from delta to omicron predominance

**Figure 8.**
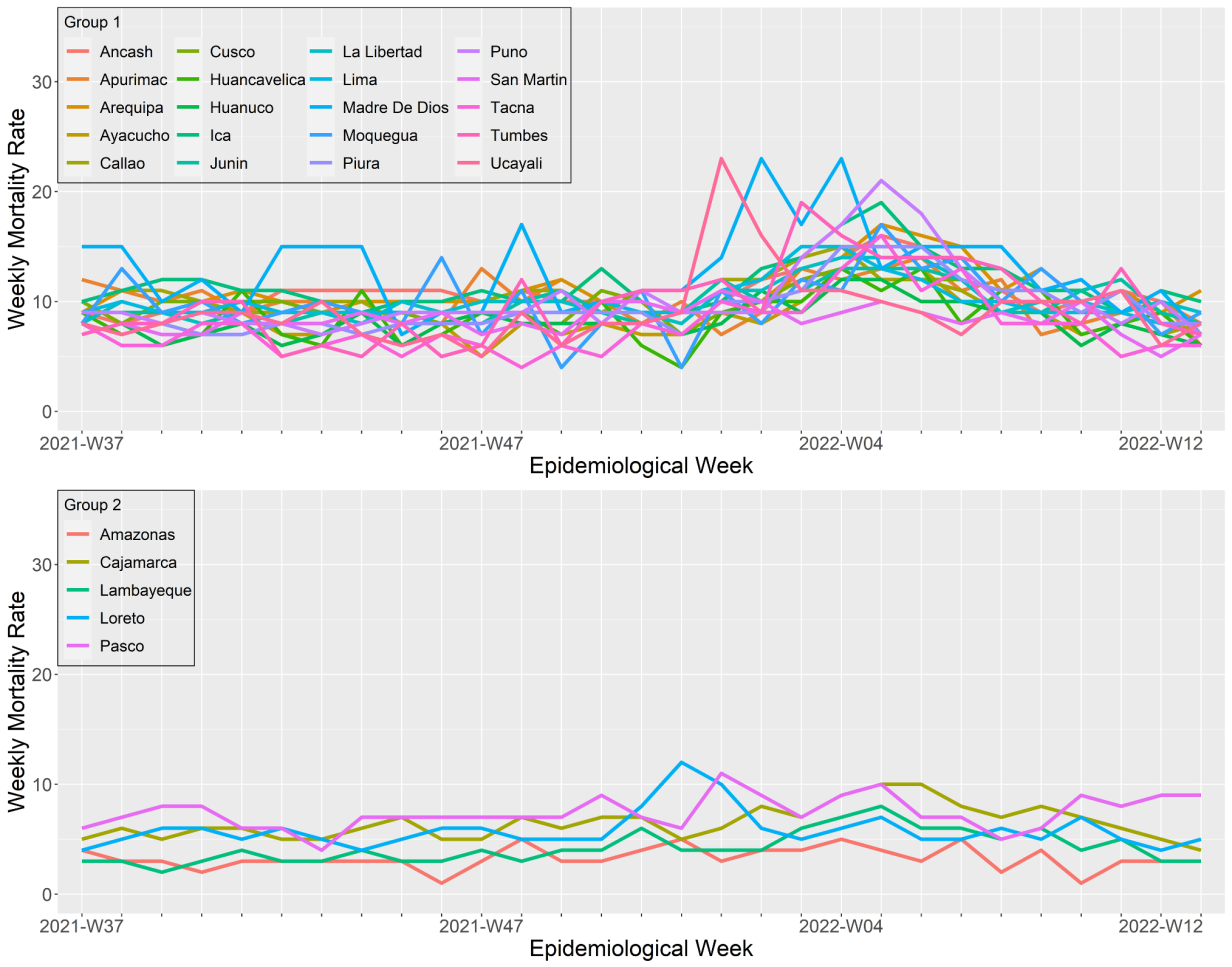
Regional wave patterns of the weekly mortality rate during the third wave of COVID-19 in Peru. Mortality is expressed as the total death counts from all causes per 100,000 inhabitants.

The third wave started when delta became the most predominant variant in Peru. However, during the third wave, omicron displaced delta and spread violently across the country, starting in most regions simultaneously. Overall, we differentiate two wave patterns ( Figure 5C and Figure 8). The first cluster included all the regions from the coast of Peru except for Lambayeque and Cajamarca, and all the regions with highlands, except for Pasco, plus the southern regions of the jungle Ucayali and Madre de Dios. Among them, Arequipa, Ancash, and Ucayali were the regions with the faster peak (4, 5, and 5 weeks, respectively); Ancash, Huánuco, La Libertad, and Tumbes were the regions with the longest waves (23, 20, 20, and 20 weeks of durations, respectively). At the same time, Madre de Dios, Ucayali, and Puno were the regions with the higher peak mortality (23, 23, and 21 deaths per 100,000 inhabitants at the wave peak, respectively). The second cluster included the regions of Amazonas, Cajamarca, Lambayeque, Loreto, Pasco, and San Martín. These waves were significantly less mortal than cluster one (mean peak mortality: 9.5 ± 2.8 vs. 16.2 ± 3.5 deaths per 100,000 inhabitants; p <0.05).

### Geographical distribution and statistical correlation between wave clusters

Geographically speaking, the spread of COVID-19 during each of Peru's waves had Lima as the epidemic epicenter and followed the coastal roads (trough the Panamericana highway) to spread to the nearby regions. During the first wave, the epidemic was reported as exported from Lima to Iquitos by over 100 Chinese tourists that traveled early in the wave, including the first case confirmed in Iquitos city ^21^. On the other side, pretty much all the regions along the Andes had the latest first waves in the country ( Figure 5A). However, with the introduction of more transmissible and infectious variants, such as alpha, gamma, and lambda, the second wave spread from the coastal regions to the highlands and jungle at once ( Figure 5B). Finally, the introduction of delta started the third wave, which spread violently with the introduction of omicron across the country, and only a few regions of the eastern jungle and northern highlands had late waves ( Figure 5C). Statistically, the first and second wave's clusters distribution correlates significantly (Spearman *rho* = 0.5468; *p* = 0.0047), but the second and third wave's clusters do not (Spearman *rho* = 0.3430; *p* = 0.0932).

## Discussion

The propagation of SARS-COV-2 variants in Peru followed different wave patterns that could be clustered in two to six wave patterns. Since the COVID-19 pandemic started in December 2019 to March 2022, Peru has been hit by three pandemic waves with different mortality rates, wave patterns, and regional clustering. The first wave was caused mainly by the index virus and spread across the 25 regions of Peru with six different wave patterns. The first wave started almost in parallel in Lima and Tumbes, supporting the hypothesis of two entry points of COVID-19 cases in Peru. Comparatively, the mortality during the first wave was nearly as high as the mortality reached during the second wave, being two of the deathlier first and second waves worldwide. The second wave was caused predominantly by the alpha and lambda variants, which were more contagious than the index virus. Both waves were substantially deathlier than the third wave, which was sparked by delta and then exploded with the predominance of omicron. The patterns of each wave varied considerably in terms of duration, peak mortality, overall mortality, and wave shapes. Still, we could identify six, two, and two different clusters of regional wave patterns during the first, second, and third waves, respectively. However, we observed some correlation between the first and second waves, which impacted the coast first and the highlands last. On the contrary, the third wave spread violently across the country, only recently hitting the remote regions of Peru with significantly lower mortality.

The Peruvian government reported ending its second wave of COVID-19 with 200,000 COVID-19 deaths ^22^; however, a recent statistical analysis estimated that by that time, Peru had accumulated around 301,000 COVID-19 deaths (95% confidence interval: 217,000–420,000) and estimated mortality of 885.6 deaths per 100,000 population (95% CI: 639.2–1234.9) ^23^. Furthermore, based on these estimates, by the end of the second wave, the COVID-19 prevalence was around 71.8%, which implied that Peru was nearly triple the prevalence estimated at the peak of the first wave (25.2%, CI95%: 22.5%–28.2%) ^24^. In our study, we calculated that during the first three waves of the COVID-19 pandemic, Peru accumulated nearly a half-million deaths from all causes and 258,106 excess deaths. We believe that all the excess deaths registered at SINADEF during the first three waves were secondary to COVID-19. The primary evidence to support that hypothesis is the perfect match between the baseline mortality and the lower weekly mortality rates after the first and third waves. This was initially observed by the Prospective Task Force ("Prospectiva"), a group of experts convened by the Peruvian government to advise and provide analytic information to the government authorities. The task force made such a hypothesis during the first wave after quantifying the excess deaths in Peru for the first time, and after, the hypothesis was validated by other government working groups during the second and third waves ^25, 26^.

The third wave in Peru seems to have started much earlier than previously reported by the Peruvian Ministry of Health ^27^. Based on our segmented regression analysis, which is a novel mathematical solution to the problem of assessing break-points in continuous data distributions ^20^, the third COVID-19 wave in Peru started in the epidemiological week 37-2021. This start date is plausible because it is the same epidemiological week where the deathlier variant delta became predominant in Peru when it surpassed the 50% threshold in the distribution of SARS-CoV-2 variants circulating in Peru (as shown in Figure 2). Also, it is plausible that the successful Peruvian COVID-19 immunization campaign mitigated the impact of delta. For context purposes, Peru started vaccinating their first-line workers and the adults 60 years old and older in February 2021, then included their high-risk adults, and then expanded the program to all adults 18 years old or over as the target population ^28^. Transition President Sagasti passed the torch to President Castillo in June 2021, with 13% of the target population covered with two doses. Soon after, in preparation for a third wave, Peru decided to accelerate the immunization campaign and set the immunization program goal to cover at least 80% of the adult population with two doses by December 2021 ^29^. To do so, Peru deployed a massive communication campaign named “I put my shoulder for Peru” (“Pongo el Hombro por el Perú”), tripled the program budget to multiply their vaccinator's brigades, and implemented the home-delivered vaccination strategy called “Let’s meet you” (“Vamos a tu encuentro”) with a territorial and community approach focused on targeting the neighborhoods with the lower vaccination coverage rates ^30^. Therefore, it is possible that, despite the predominance of delta, Peru controlled its impact by accelerating its COVID-19 immunization campaign and progressively achieving its programmatic goal of a 50% coverage in October 2021 ^31^ and 80% in December 2021 ^32^, before omicron became the predominant variant (as shown in Figure 2).

Another important observation from our study is that the third wave not only registered a substantially reduced mortality compared to previous waves, but also registered a substantial reduction in the fraction that excess deaths represent from the total death count from all causes, compared to the first and second wave (47% vs. 60% and 65%, respectively). Furthermore, we also observed an increased mortality ratio among adults 60 years old and older and the mortality among adults 20- to 59-year-old in the third wave relative to the first and second wave (to 16.9 from 13.1 to 13.4, respectively). These results add to the evidence that highlights the impact of COVID-19 vaccination in reducing COVID-19 mortality, but at the same time the increased mortality due to non-COVID-19 causes in the latest months. With most efforts focused on responding to the COVID-19 pandemic, low-middle income countries like Peru have neglected their resources to prevent common non-COVID-19 causes of death, such as those caused by cancer or other preexisting chronic diseases. Scientists from the US ^33^, Germany ^34^ and Italy ^35^ reported increased mortality in older people during the pandemic and underlying conditions such as heart, kidney, liver, and lung chronic diseases, diabetes, hypertension, dementia, and immunological diseases with and without COVID-19. Hence, in a scenario where the effect of the vaccine reduces COVID-19 mortality, it is expected that non-COVID-19 causes of death became the leading causes of death if it is true that most of the excess deaths were, in fact, secondary to COVID-19 in Peru.

In context, Peru shifted from a first wave caused by the ancestor variant, with a basic reproductive number estimated for the country as 2.97 and in Lima, the capital and only megacity of Peru, as 2.88 ^36^. This means that the average number of infected contacts per infected individual was close to three, but more importantly three contacts without previous exposure to SARS-CoV-2. Hence, COVID-19 spread quickly across urban areas, hitting clustered cities like Iquitos hardly, where 70% of its total population got COVID-19 with a considerable cost in lives ^8^. Then, Peru faced its most deathly wave caused by different and more contagious SARS-CoV-2 variants, including lambda, gamma, and delta, with anecdotic cases of alpha and mu ^37^. In the timeline, during the second wave, the SARS-CoV-2 variants predominance shifted from Lambda predominance, which competed with gamma and displaced the ancestor variant completely ^38^, towards delta predominance, which was first reported downhill in the second wave and along its end completely displaced Lambda and the other SARS-CoV-2 variants prior. In the third wave, omicron violently spread across the country, completely displacing delta in one month, starting and predominating along with the whole wave.

The key strength of our study is that we analyzed big data from a very reliable death registration system and contrasted three very different COVID-19 waves with a good sense of which SARS-CoV-2 variants dominated each wave. Furthermore, the study might be overpowered because the high mortality observed across each wave allowed us to characterize each pattern properly. Another critical observation that allowed us to simplify the interpretation of our results was the homogenous population in terms of age groups and gender, which allowed us to avoid the need to use standardized mortalities, which was our original intention. We took advantage of this license to simplify our analysis and communicate our results in the same terms as commonly reported by the Peruvian government. However, it is essential to highlight that although the regional populations seem comparable in age groups and gender distribution, there is a crucial variability in rurality, poverty, sanitation, race, altitude, and population density that requires further analysis. Additionally, it is essential to mention that although SINADEF is described as reliable, the register was four years old in 2020 and had 75% real-time data entry, with a remaining 25% having a two to four weeks digitalization delay ^39^. Also, it is possible that SINADEF sub-registers the deaths at the peak of each wave due to personal shortages and unregistered burials, particularly in the rural areas. This is important because the analysis in our study was restricted to mortality data rather than COVID-19 case incidence.

## Conclusion

Based on the results, we can affirm that Peru's first, second, and third COVID-19 waves were substantially different at the national and regional levels. These differences were more noticeable in the first wave, which showed six different wave patterns with distinct beginnings, mortality rates, mortality peaks, and duration. Although it proved to be deathlier, the second wave was more homogeneous than the first wave. The third wave, on the contrary, was the shortest and less mortal wave, with a homogeneous but certainly more explosive spread than the second wave. The cluster analysis showed that the second and third waves at the regional level could be grouped into two large groups with different mortality peaks. Likewise, it is necessary to mention that the geographical factor would also be a possible explanation for the behavior of the epidemiological waves because neighboring regions were consistently grouped in the same groups. We consider that future research should address this topic in greater depth.

## Data Availability

The data used in our study is open data curated by the Peruvian government and freely available from ( Table 1): https://www.datosabiertos.gob.pe/dataset/poblaci%C3%B3n-peru https://www.datosabiertos.gob.pe/dataset/informaci%C3%B3n-de-fallecidos-del-sistema-inform%C3%A1tico-nacional-de-defunciones-sinadef-ministerio https://web.ins.gob.pe/es/covid19/secuenciamiento-sars-cov2 https://geoservidorperu.minam.gob.pe/geoservidor/archivos/download/Limite_departamental.rar The official administrative boundaries for Peru regions are owned by the Ministry of Environment and can be accessed through the website https://www.geogpsperu.com.
