# Supplementary material for: Regional clustering and waves patterns due to COVID-19 by the index virus and the lambda/gamma, and delta/omicron SARS-CoV-2 variants in Peru
Source: Gates Open Res. 2023 Nov 20;6:74. Originally published 2022 Jul 1. [Version 2] doi: 10.12688/gatesopenres.13644.2 (PMC10692151; doi:10.12688/gatesopenres.13644.2)
Supplement: Peer review history

# Reviewer report: Reviewer response for version 2

**DOI:** 10.21956/gatesopenres.16482.r35520

**Published:** 2023-12-01

**Authors:** Gerardo Santos-Lopez

**Affiliations:** 1 Instituto Mexicano del Seguro Social, Puebla, Mexico

The authors have corrected the article. I have no further comments to make.

Is the work clearly and accurately presented and does it cite the current literature?

Yes

If applicable, is the statistical analysis and its interpretation appropriate?

Yes

Are all the source data underlying the results available to ensure full reproducibility?

Yes

Is the study design appropriate and is the work technically sound?

Yes

Are the conclusions drawn adequately supported by the results?

Yes

Are sufficient details of methods and analysis provided to allow replication by others?

Yes

Reviewer Expertise:

Diagnosis and epidemiology of viral infections, viral pathogenesis and development of antivirals.

I confirm that I have read this submission and believe that I have an appropriate level of expertise to confirm that it is of an acceptable scientific standard.

# Reviewer report: Reviewer response for version 2

**DOI:** 10.21956/gatesopenres.16482.r35521

**Published:** 2023-11-23

**Authors:** Anne Bozack

**Affiliations:** 1 Stanford University, Stanford, California, USA

I thank the authors for their response to the reviewers' comments and edits to the manuscript. The authors have sufficiently addressed my comments.

Is the work clearly and accurately presented and does it cite the current literature?

Yes

If applicable, is the statistical analysis and its interpretation appropriate?

Yes

Are all the source data underlying the results available to ensure full reproducibility?

Yes

Is the study design appropriate and is the work technically sound?

Yes

Are the conclusions drawn adequately supported by the results?

Yes

Are sufficient details of methods and analysis provided to allow replication by others?

Yes

Reviewer Expertise:

Environmental health science and molecular epidemiology

I confirm that I have read this submission and believe that I have an appropriate level of expertise to confirm that it is of an acceptable scientific standard.

# Reviewer report: Reviewer response for version 1

**DOI:** 10.21956/gatesopenres.14925.r35208

**Published:** 2023-11-08

**Authors:** Anne Bozack

**Affiliations:** 1 Stanford University, Stanford, California, USA

Overall, this manuscript provides a comprehensive and straight-forward analysis of the COVID-19 pandemic in Peru based on temporal mortality waves and regional clustering. This study contributes to better understanding the origin and progression of the pandemic in the country. Below, I note sections that would benefit from additional details as well as several typos that should be corrected.

- Statistical methods: I believe the elbow and silhouette plots are based on K-means clustering. The authors should specify this as well as which variables are included in the cluster analysis. 
- Results, first section: "37-2022 to the 13-2022" should be "37-202 **1** to the 13-2022".
- Results, first section: I believe the word "coincidently" is not being accurately used in this context, since the authors define the division of waves and pandemic period based on the points of lowest mortality. I suggest using the word "consequently" instead.
- Discussion: In the study's limitations, the authors should note that the analysis was restricted to mortality data, rather than looking and Covid incidence. 
- Results, geographical distribution: " *p* = 0047" is missing a decimal point.
- Figure 2: The legend is not correct.
- Figure 4: "(E)" needs to be reformatted.

Is the work clearly and accurately presented and does it cite the current literature?

Yes

If applicable, is the statistical analysis and its interpretation appropriate?

Yes

Are all the source data underlying the results available to ensure full reproducibility?

Yes

Is the study design appropriate and is the work technically sound?

Yes

Are the conclusions drawn adequately supported by the results?

Yes

Are sufficient details of methods and analysis provided to allow replication by others?

Yes

Reviewer Expertise:

Environmental health science and molecular epidemiology

I confirm that I have read this submission and believe that I have an appropriate level of expertise to confirm that it is of an acceptable scientific standard, however I have significant reservations, as outlined above.

# Reviewer report: Reviewer response for version 1

**DOI:** 10.21956/gatesopenres.14925.r34921

**Published:** 2023-09-25

**Authors:** Gerardo Santos-Lopez

**Affiliations:** 1 Instituto Mexicano del Seguro Social, Puebla, Mexico

The manuscript presented by Toyama et al. provides a comprehensive analysis of the three waves of COVID-19 in Peru. It covers a wide range of topics, from fundamental data to spatial and temporal distribution analyses across regions and provinces. The document is well-crafted, offering detailed descriptions, thorough analysis, and robust statistical modeling of the data. I've noticed a few minor issues that can be easily resolved:

- There is an issue with the description in Figure 2; it really refers to Figure 3.
- On page 7, in the "Regional cluster analysis" section, it mentions "(Figure 4C and Figure 4D)," but it should refer to Figures 4E and 4F.
- I recommend highlighting the highest mortality rates in Table 3, in line with what was mentioned in the results section regarding the "Variability of the mortality rate across regions by wave, age, and gender."
- Please review the wording in the description of Figure 4. There appear to be errors related to sections A-F.

Is the work clearly and accurately presented and does it cite the current literature?

Yes

If applicable, is the statistical analysis and its interpretation appropriate?

Yes

Are all the source data underlying the results available to ensure full reproducibility?

Yes

Is the study design appropriate and is the work technically sound?

Yes

Are the conclusions drawn adequately supported by the results?

Yes

Are sufficient details of methods and analysis provided to allow replication by others?

Yes

Reviewer Expertise:

Diagnosis and epidemiology of viral infections, viral pathogenesis and development of antivirals.

I confirm that I have read this submission and believe that I have an appropriate level of expertise to confirm that it is of an acceptable scientific standard, however I have significant reservations, as outlined above.
